# The neural bases of spatial frequency processing during scene perception

**DOI:** 10.3389/fnint.2014.00037

**Published:** 2014-05-07

**Authors:** Louise Kauffmann, Stephen Ramanoël, Carole Peyrin

**Affiliations:** ^1^University Grenoble AlpesLPNC, Grenoble, France; ^2^CNRS, LPNC, Université Pierre Mendès FranceGrenoble, France

**Keywords:** natural scene, spatial frequencies, coarse-to-fine, hemispheric specialization, retinotopy, parahippocampal place area

## Abstract

Theories on visual perception agree that scenes are processed in terms of spatial frequencies. Low spatial frequencies (LSF) carry coarse information whereas high spatial frequencies (HSF) carry fine details of the scene. However, how and where spatial frequencies are processed within the brain remain unresolved questions. The present review addresses these issues and aims to identify the cerebral regions differentially involved in low and high spatial frequency processing, and to clarify their attributes during scene perception. Results from a number of behavioral and neuroimaging studies suggest that spatial frequency processing is lateralized in both hemispheres, with the right and left hemispheres predominantly involved in the categorization of LSF and HSF scenes, respectively. There is also evidence that spatial frequency processing is retinotopically mapped in the visual cortex. HSF scenes (as opposed to LSF) activate occipital areas in relation to foveal representations, while categorization of LSF scenes (as opposed to HSF) activates occipital areas in relation to more peripheral representations. Concomitantly, a number of studies have demonstrated that LSF information may reach high-order areas rapidly, allowing an initial coarse parsing of the visual scene, which could then be sent back through feedback into the occipito-temporal cortex to guide finer HSF-based analysis. Finally, the review addresses spatial frequency processing within scene-selective regions areas of the occipito-temporal cortex.

## INTRODUCTION

Scenes containing more realistic and more natural stimuli have increasingly become the object of scientific interest over the last 20 years, as they involve the perception of stimuli which are more complex and more realistic than simple objects or drawings. It is now widely agreed that visual recognition of scenes is a fast, automatic and reliable process. Experimental studies have shown that complex natural scenes can be categorized very rapidly (under 150 ms; Thorpe et al., 1996), indicating that a simple and efficient coding process is involved. There is considerable evidence suggesting the importance of the spatial frequency contents of images during scene recognition (Ginsburg, 1986; Field, 1987; Tolhurst et al., 1992; Hughes et al., 1996). On one hand, the primary visual cortex is mainly dominated by complex cells which respond preferentially to spatial frequencies (Poggio, 1972; De Valois et al., 1982a,b). On the other hand, findings from simulation and psychophysical experiments indicate that information from low/medium frequencies of the amplitude spectrum suffices to enable scene categorization (Torralba and Oliva, 2003; Guyader et al., 2004). Supported by convergent data from the functional neuroanatomy of magnocellular and parvocellular visual pathways (Van Essen and Deyoe, 1995), neurophysiological recordings in primates (for a review, see Bullier, 2001), and psychophysical results in humans (Ginsburg, 1986; Hughes et al., 1996), current influential models of visual perception (Schyns and Oliva, 1994; Bullier, 2001; Bar, 2003; Hegde, 2008) suggest that the first stage of visual analysis consists of the extraction of visual elementary features at different spatial frequencies. Low spatial frequencies (LSF), conveyed by fast magnocellular pathways, provide a coarse information about a visual stimulus (e.g., the global shape and structure of a scene), whereas high spatial frequencies (HSF), conveyed more slowly by the parvocellular pathways, provide finer information about the stimulus (e.g., the edges and borders of an object in the scene).

However, exactly how and where spatial frequencies are processed within the brain remain unsettled questions. The debate on retinotopic organization and/or the existence of cerebral asymmetries in the occipital cortex in spatial frequency processing is still ongoing in the literature. A number of studies demonstrated a retinotopic mapping of spatial frequency processing in the occipital cortex and have for example showed that the perception of HSF sinusoidal gratings activated the foveal representation in all retinotopic areas of the occipital cortex, and LSF sinusoidal gratings activated more peripheral representations in the same cortical areas (Sasaki et al., 2001; Henriksson et al., 2007). However, other authors argue in favor of the hemispheric specialization for spatial frequency processing at the level of visual retinotopic areas, with the right hemisphere preferentially specialized in the processing of LSF information and the left hemisphere preferentially specialized in HSF information processing (Iidaka et al., 2004; Peyrin et al., 2004, 2006). It appears therefore important to investigate both retinotopic processing and hemispheric specialization on the same visual stimuli.

**FIGURE 1:**
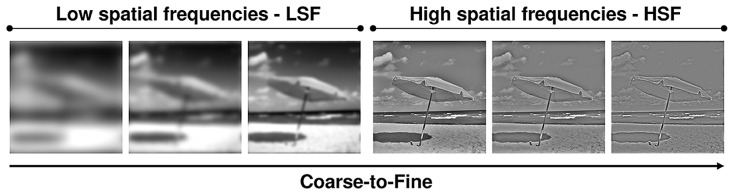
**Coarse-to-fine sequence of spatial frequency processing (from low-to-high spatial frequencies) during scene perception**.

In addition, there is considerable evidence suggesting that spatial frequency processing takes place in a predominantly and default coarse-to-fine sequence (**Figure 1**) However, the cerebral circuit of the coarse-to-fine perception of scenes has never been investigated in humans. On the basis of neurophysiological recordings in nonhuman primates, Bullier (2001) suggested that during perception of a scene, LSF which are conveyed more rapidly than HSF by fast magnocellular pathways, access the occipital cortex and high-order cortical areas in the dorsal cortical stream (parietal and frontal cortices) allow a coarse perceptual parsing of the visual input, prior to their complete propagation along the ventral cortical stream (inferotemporal cortex) which ultimately mediates the input recognition. The initial low-pass analysis would serve to refine the subsequent processing of HSF, conveyed more slowly by parvocellular pathways through the ventral cortical stream. It is now essential to match data from non human primates with human data. Finally, the ventral visual stream contains a mosaic of different areas that respond selectively to different categories of visual stimuli (Haxby et al., 2001; Lerner et al., 2001; Spiridon and Kanwisher, 2002). While several studies (Epstein and Kanwisher, 1998; Bar and Aminoff, 2003; Bar, 2004; Epstein, 2005, 2008; Aminoff et al., 2007; Epstein and Higgins, 2007; Dilks et al., 2013) agree that a prominent region in the inferotemporal cortex known as the parahippocampal place area (PPA), the retrosplenial cortex (RSC) and a region around the transverse occipital sulcus called the occipital place area (OPA) all play a major role in the perception of scenes in humans, the specific functions supported by scene-selective regions during the spatial frequency processing in scenes remain unclear.

The present review addresses these issues and aims to identify the cerebral regions differentially involved in low and high spatial frequency processing and to clarify their attributes during scene perception.

## NEURAL CORRELATES OF SPATIAL FREQUENCY PROCESSING DURING SCENE PERCEPTION

Many authors postulate that the two cerebral hemispheres are differently involved in spatial frequency processing, the right hemisphere predominating in the processing of LSF, and the left hemisphere predominating in the processing of HSF. Cerebral asymmetries have been observed in behavioral studies conducted on healthy participants (Sergent, 1982, 1983; Sergent and Hellige, 1986; Kitterle et al., 1990, 1992; Chokron et al., 2003; Peyrin et al., 2003), in neurological patients (Robertson et al., 1988; Lamb et al., 1990; Robertson and Lamb, 1991; Peyrin et al., 2006; Dos Santos et al., 2013), and from functional neuroimaging studies (Fink et al., 1996, 2000; Martinez et al., 1997, 2001; Heinze et al., 1998; Kenemans et al., 2000; Mangun et al., 2000; Yamaguchi et al., 2000; Wilkinson et al., 2001; Han et al., 2002; Iidaka et al., 2004; Lux et al., 2004; Peyrin et al., 2004; Weissman and Woldorff, 2005; Musel et al., 2013). However, the hemispheric specialization for spatial frequency processing was largely inferred from studies assessing cerebral asymmetries during the processing of global and local information.

### PSYCHOPHYSICAL ARGUMENTS FOR HEMISPHERIC SPECIALIZATION IN SPATIAL FREQUENCY PROCESSING

**FIGURE 2:**
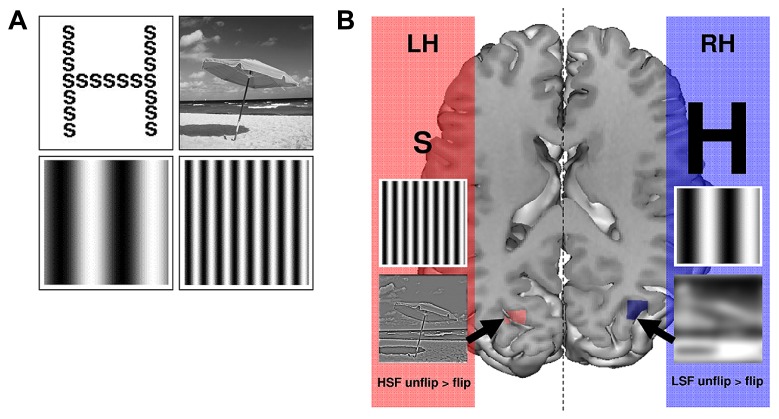
**(A)** Example of stimuli used to assess cerebral asymmetries for spatial frequencies. Top-left: Hierarchical visual forms, consisting of a large global letter made up of small local letters; Top-right: Scene; Bottom-left: low spatial frequency (LSF) sinusoidal grating; Bottom-right: high spatial frequency (HSF) sinusoidal grating. **(B)** Hemispheric specialization: the left hemisphere (LH) is predominantly involved in the local letter identification, HSF grating identification and HSF categorization; the right hemisphere (RH) is predominantly involved in the global letter identification, LSF grating identification and LSF categorization. Activations reported showed stronger activation in the left than the right occipital cortex for HSF categorization [(HSF unflip > flip) contrast] and stronger activation in the right than the left occipital cortex for LSF categorization [(LSF unflip > flip) contrast]. Figure adapted from Peyrin et al. (2004).

The first experimental evidence in support of hemispheric specialization for global and local processing comes from psychophysical studies using hierarchical forms as visual stimuli (i.e., in general a large global letter made up of small local letters; Navon, 1977; Kinchla and Wolfe, 1979; **Figure 2A**). Using hierarchical visual stimuli displayed in either the left visual field (projecting directly to the right hemisphere) or the right visual field (projecting directly to the left hemisphere), Sergent (1982) demonstrated that the identification of the global letter was faster when displayed in the left visual hemifield/right hemisphere, and that the identification of local letters occurred more rapidly when they were displayed in the right visual hemifield/ left hemisphere. These results suggest a right hemispheric specialization for the processing of global information, and a left hemispheric specialization for the processing of local information. Based on evidence that global information is predominantly conveyed by LSF, and that local information is predominantly conveyed by HSF (Schulman et al., 1986; Badcock et al., 1990; Lamb and Yund, 1993), the cerebral asymmetries observed during global and local processing have been interpreted as reflecting the hemispheric specialization for LSF and HSF processing, respectively (Sergent, 1982).

However, the relationship between global and local information, and LSF and HSF, respectively, is far from univocal within hierarchical visual forms (Palmer, 1993). It is for example possible that global information is conveyed by both LSF and HSF. Hemispheric specialization for spatial frequency processing was therefore subsequently tested by directly manipulating the spatial frequency content of visual stimuli, using either sinusoidal gratings (Kitterle et al., 1990, 1992; Kitterle and Selig, 1991; **Figure 2A**) or scene images (Peyrin et al., 2003, 2006; **Figure 2A**). It should be noted that this type of manipulation is not feasible with hierarchical forms because low-pass filtering cancels out the local form and renders the task impossible. We evaluated hemispheric asymmetry in healthy participants in a series of psychophysical studies (Peyrin et al., 2003, 2006), by making explicit changes in the spatial frequency spectrum of scene images, which were displayed in either the left or the right visual fields. In the initial study, participants were asked to recognize a target scene (a city or a highway) filtered in either LSF or HSF (Peyrin et al., 2003). Results showed more rapid recognition of LSF scenes when they were displayed in the left visual hemifield/right hemisphere than when they were presented in the right visual field/left hemisphere. Conversely, recognition of HSF scenes occurred more rapidly in the right visual hemifield/left hemisphere than the left visual hemifield/right hemisphere. This study demonstrated a right hemispheric predominance for LSF and a left hemispheric predominance for HSF processing. It should be noted that the hemispheric specialization in question has been observed in males, but not in females (Peyrin et al., 2006). These results are consistent with studies showing a lesser degree of lateralization in female functional cerebral organization compared to males (McGlone and Kertesz, 1973; Voyer, 1996). Certain factors of interference, which may affect processing speed may render detection of hemispheric specialization in healthy females more difficult. For example, the hormonal level fluctuations over the menstrual cycle has been evidenced to modulate hemispheric asymmetries in visual, attentional, and language processes (Hausmann and Güntürkün, 2000; Hausmann et al., 2002; Hausmann, 2005), and to affect interhemispheric transfer time (Hausmann et al., 2013).

### NEURAL CORRELATES OF HEMISPHERIC SPECIALIZATION IN SPATIAL FREQUENCY PROCESSING

Neuropsychological and neuroimaging studies that use hierarchical visual forms provide conflicting evidence on which cortical structures present hemispheric specialization. Robertson et al. (1988) showed impairment in the performance of tasks involving the perception of hierarchical visual form in patients with unilateral damage to the temporo-parietal junction. Performance of patients with a lesion situated in the left superior temporal cortex was impaired during the identification of local elements, whereas patients suffering from lesions in the right temporo-parietal junction exhibited poor performance during the identification of the global form. These data suggests the right temporo-parietal junction specialization for global processing, and the left temporo-parietal junction specialization for local processing. However, using positron emission tomography, Fink et al. (1996; see also Fink et al., 1997, 2000) reported cerebral asymmetries at a lower level of visual cortical processing, with a right lingual gyrus activation during the identification of the global form and a left inferior occipital gyrus activation during the identification of local elements. Using event-related brain potentials (ERPs), Heinze et al. (1998; see also Mangun et al., 2000) failed to show hemispheric specialization in the first-stage of the visual analysis. Instead, their results show long latency asymmetries (260–360 latency range) for global and local processing, suggesting that cerebral asymmetries was rather present at the higher-stage of the visual analysis. Some functional imaging data have, furthermore, revealed an attentional cortical mechanism located in the temporo-parietal junction which controls the attentional selection of information presented either at global or the local level depending on the visual task demands (Robertson et al., 1988; Robertson and Lamb, 1991; Fink et al., 1996; Yamaguchi et al., 2000; Wilkinson et al., 2001; Weissman and Woldorff, 2005). For example, Yamaguchi et al. (2000) recorded ERPs while participants shifted their attention to the global or local level of hierarchical visual forms (the shift direction was controlled by a cue preceding the stimulus). Cerebral asymmetries were observed during the global and local processing of hierarchical forms, but also during the time interval of attention directed toward global or local levels by the cues. ERP responses indicated greater right-hemisphere amplitudes located in the right temporo-parietal junction when attention was directed at global level, and greater left-hemisphere amplitudes located in the left temporo-parietal junction when it was directed at local level. This study provided a neural basis for a “top-down” mechanism of allocation of attention to global and local information, and revealed the asymmetrical involvement of the temporal-parietal regions.

Neuroimaging studies previously mentioned have provided conflicting results concerning hemispheric specialization for spatial frequency processing using hierarchical visual forms as stimuli. Subsequent studies, including those of our own team, which involved the direct manipulation of the spatial frequency content of stimuli, provided evidence of hemispheric specialization involving occipital areas (Iidaka et al., 2004; Peyrin et al., 2004). In an fMRI study, Peyrin et al. (2004) investigated the hemispheric specialization for spatial frequency processing during the recognition of LSF and HSF scenes (city vs. highway scenes at a visual angle of 4°). Comparison of LSF to HSF scene recognition, revealed significant activation in regions which are known to be involved in scene processing: the right anterior temporal region which is particularly sensitive to familiar versus unfamiliar scenes (Nakamura et al., 2000), and the right parahippocampal gyrus which is known to be involved in tasks requiring the retrieval of topographical information in scenes (Maguire et al., 1998; it should be noted that right-side parahippocampal gyrus activation did not correspond to PPA activation reported by Epstein and Kanwisher, 1998). These results suggest that in Peyrin et al. (2004), scene perception was based mainly on LSF extraction and analysis, and they support the models proposing the prevalence of LSF information in scene categorization (coarse-to-fine strategy; Schyns and Oliva, 1994). Significant activation also occurred in the right inferior parietal lobule near the temporo-parietal junction. This activation was interpreted as reflecting an attentional control mechanism during spatial frequency selection. Yamaguchi et al. (2000) had previously shown cerebral activity in the right temporo-parietal area for a global attention shift during the perception of hierarchical letter forms (i.e., allocation of attention to global information). Finally, LSF scene recognition (as opposed to HSF) activated the superior temporal cortex bilaterally. This particular result concerned us, because it contradicted neuropsychological studies (Robertson et al., 1988; Lamb et al., 1990; Robertson and Lamb, 1991), showing specialization of the right superior temporal cortex in the perceptual processing of global information (supposed to be preferentially conveyed by LSF), and specialization of the left superior temporal cortex in the perceptual processing of local information (supposed to be preferentially conveyed by HSF). It should be noted that HSF scene recognition (as opposed to LSF) failed to show significant activation, suggesting a processing bias toward LSF.

**FIGURE 3:**
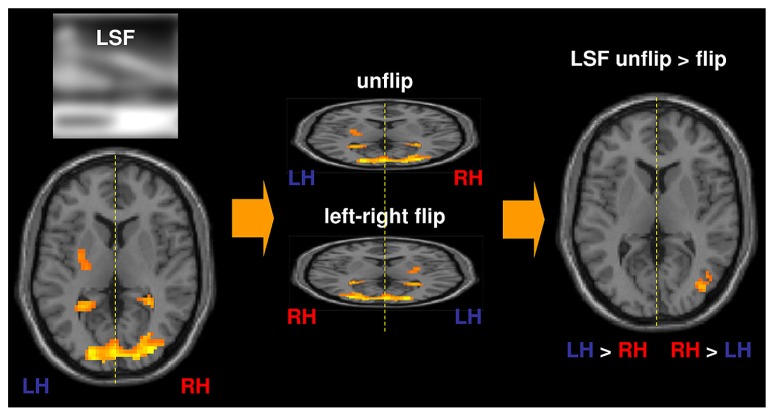
**Method of direct inter-hemispheric comparison.** Two sets of functional volumes, obtained from functional scans, are compared at individual level. One set is represented by functional volumes in accordance with neurological convention (the left hemisphere – LH appears on the left side of images) and the other set is represented by the same functional volumes this time in accordance with radiological convention (the right hemisphere – RH appears on the left side of images). Images from the second set were “flipped” by 180° in the midsagital plane, thus providing “mirror” images of the first set. Contrasts between “unflipped” and “left-right flipped” images were then calculated for each of the spatial frequency components of natural scenes. In order to assess hemispheric predominance during the perception of LSF scenes, for instance, the following contrast was calculated: LSF unflip > flip. Regions which were more highly activated in the left hemisphere than in the right hemisphere appear on the left side, and regions which were statistically more highly activated in the right hemisphere than in the left hemisphere appear on the right side.

Based on behavioral studies in which performances between the two visual hemifields are directly compared (see our above-mentioned original psychophysical experiments; Sergent, 1982; Kitterle et al., 1990, 1992; Kitterle and Selig, 1991; Peyrin et al., 2003, 2006), we suggested to directly compare activation between the two hemispheres in order to assess cerebral asymmetries in fMRI study. For this purpose, we created an fMRI method of direct inter-hemispheric comparison. Two sets of functional volumes, obtained from functional scans, are compared at individual level. One set is represented by functional volumes in accordance with neurological convention (the left hemisphere appears on the left side of images) and the other set is represented by the same functional volumes this time in accordance with radiological convention (the right hemisphere appears on the left side of images). Images from the second set are “flipped” by 180° in the midsagital plane, thus providing “mirror” images of the first set. Contrasts between “unflipped” and “left-right flipped” functional volumes from the same experimental condition allow to compare activity in one hemisphere with activity in homologous regions of the other hemisphere (Iidaka et al., 2004; Peyrin et al., 2004, 2005; Musel et al., 2013; see also Cousin et al., 2006 for an application of this method on language processes; **Figure 3**). This method revealed greater activation in the right than the left middle occipital gyrus for LSF scene recognition, and greater activation in the left than the right middle occipital gyrus for HSF scene recognition (**Figure 2B**). This study provided new evidence for hemispheric specialization at the first cortical level of visual analysis. Analyzing fMRI data with a more traditional approach which contrasts spatial frequencies to one another, we observed a higher degree of activation for LSF scenes (as opposed to HSF), while the reverse contrast did not reveal any significant activation. This study suggests that the results considerably differ according to the method applied to analysis fMRI data. Inter-hemispheric comparison seems more appropriate for the investigations of cerebral asymmetries, since it allows any main effect deriving from spatial frequency bias to be canceled out.

We proceeded to investigate the role of the occipital cortex in spatial frequency processing using a neuropsychological approach (Peyrin et al., 2006). We studied the categorization of LSF and HSF scenes in a female neurological patient who suffered from a focal lesion in the right occipito-temporal cortex following the embolization of an arterioveinous malformation. This lesion had induced a left homonymous hemianopsia. Two evaluations were conducted, the first 1 week prior to surgical intervention and the second 6 months afterward. As expected, the performance of the patient was more severely impaired for LSF than HSF scene recognition following embolization. This result suggests again the right occipital cortex specialization for LSF, and on a more general level suggests that hemispheric specialization could occur in women, although this is difficult to demonstrate behaviorally in the healthy population. This finding highlights the necessity of studying males and females together and both normal and brain-damaged patients’ performance in order to establish the neural correlates of visual functions.

The extent to which hemispheric asymmetries during spatial frequency processing result from perceptual or attentional processes remains to be determined. While some studies have clearly demonstrated that attentional processes exert control on hemispheric specialization in the processing of global and local information at high-level stages of visual processing (e.g., via the temporo-parietal junction; Robertson et al., 1988; Robertson and Lamb, 1991; Fink et al., 1996; Heinze et al., 1998; Yamaguchi et al., 2000; Wilkinson et al., 2001; Weissman and Woldorff, 2005), other studies have evidenced hemispheric asymmetries at lower-level stages, in the occipital cortex (Fink et al., 1997, 2000; Peyrin et al., 2004; Musel et al., 2013). However, activation of the occipital cortex was frequently associated with activation of the temporo-parietal junction in these studies. This cortical structure may have exerted attentional influence on lower-level areas. Furthermore, a number of neuroimaging studies have evidenced attentional modulation of activity in early visual areas (Tootell et al., 1998; Watanabe et al., 1998; Brefczynski and DeYoe, 1999; Gandhi et al., 1999; Martinez et al., 1999; Sasaki et al., 2001; Silver et al., 2007; Saygin and Sereno, 2008). For example, Martinez et al. (1999) showed that attending to a target whose location was cued by an arrow enhanced the amplitude of activation in striate and extrastriate visual areas. Cerebral asymmetries observed at low-level stages of visual processing, such as the occipital cortex, may not, therefore, necessarily result from strictly perceptual processes.

However, despite the considerable body of research in favor of the hemispheric specialization for spatial frequency processing in the occipital cortex, other authors postulate that a spatial frequency processing mapping according to the retinotopic organization of the visual cortex.

### RETINOTOPIC PROCESSING OF SPATIAL FREQUENCIES

**FIGURE 4:**
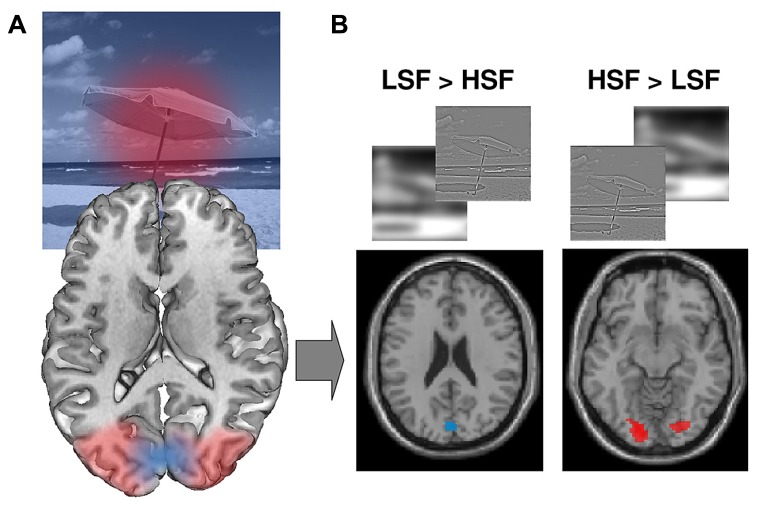
**(A)** Retinotopic mapping of the visual field on the visual cortex. The central (foveal) part of the visual field is represented at the very back of the visual cortex and laterally. More peripheral regions of the visual field are represented further forward in the medial part of the visual cortex. **(B)** Retinotopic organization of spatial frequency processing during scene perception: LSF [as opposed to HSF, (LSF > HSF) contrast] scene categorization recruits areas dedicated to peripheral vision, while HSF [as opposed to LSF, (LSF > HSF) contrast] scene categorization recruits areas dedicated to foveal vision. Figure adapted from Musel et al. (2013).

Imaging data obtained from patients with cerebral lesions (Holmes, 1918; Horton and Hoyt, 1991) and from healthy participants (Engel et al., 1994, 1997) show that the human primary visual cortex is retinotopically organized. The central (foveal) part of the visual field is represented at the very back of the visual cortex and more peripheral regions of the visual field are represented further forward (**Figure 4A**). Importantly, the distribution of retinal photoreceptors and retinal ganglion cells is nonhomogeneous throughout the retina (Curcio and Allen, 1990; Curcio et al., 1990). The density of cones and midget ganglion cells from which the parvocellular pathway originates and which are used to process HSF information, is greatest in the fovea, while the density of rods and parasol ganglion cells from which the magnocellular pathway originates and which are used to process LSF information, increases with foveal eccentricity. Therefore HSF information could be predominantly processed in the areas dedicated to foveal vision. Similarly, LSF information might well be predominantly processed in the areas devoted to peripheral vision.

Neurophysiological studies performed on cats (Everson et al., 1998; Issa et al., 2000), primates (De Valois et al., 1982a; Foster et al., 1985; Tootell et al., 1988; Gegenfurtner et al., 1997; Xu et al., 2007) and humans (Singh et al., 2000; Sasaki et al., 2001; Henriksson et al., 2007) have mapped the representation of spatial frequencies in retinotopic areas. In an fMRI study, using retinotopic encoding with achromatic sinusoidal gratings, Sasaki et al. (2001) showed that LSF were mapped on the peripheral visual field representation of the occipital cortex, whereas HSF were mapped on the central visual field representation. More recently, Henriksson et al. (2007) evidenced that in the retinotopic area of the occipital cortex, lower spatial frequencies selectivity was observed as eccentricity of the achromatic sinusoidal grating increased. Other studies have provided evidence of consistent cortical retinotopic mapping of more complex cognitive functions, such as visual spatial attention (Tootell et al., 1998; Watanabe et al., 1998; Brefczynski and DeYoe, 1999; Gandhi et al., 1999; Martinez et al., 1999; Sasaki et al., 2001; Silver et al., 2007) and working memory (Pratte and Tong, 2014) in the early visual areas, as well as in the higher cortical areas, such as in the temporal, parietal, and frontal cortices (Silver et al., 2005; Hagler and Sereno, 2006; Wandell et al., 2007; Saygin and Sereno, 2008; Arcaro et al., 2009, 2011; Sheremata et al., 2010). Tootell et al. (1998), for example, showed that paying attention to a specific location in the visual field increased activity in the corresponding retinotopic location of the extrastriate visual areas. Attentional modulation which was similar, albeit to a lesser degree, was also observed in the primary visual cortex (V1). Saygin and Sereno (2008) subsequently investigated the independent modulation of retinotopic responses by visual stimulus properties and attention in a number of areas exhibiting retinotopic organization (in the occipital cortex, the precuneus, the motion-sensitive temporal cortex, the intraparietal sulcus, and the frontal eye fields in the frontal cortex). These authors used retinotopically rotating polar angle mapping with point-light biological motion figures as complex visual stimuli. Participants fixated and viewed a rotating pie-shaped wedge containing biological motion figures. In the background, biological motion figures were either surrounded by either scrambled figures (stimulus contrast) or similar figures (no stimulus contrast). Participants were asked to perform one of two tasks while fixating – they were asked to attend to either the wedge (attention) or to the center of gaze (no attention). The authors demonstrated that the retinotopy of early visual areas was mainly driven by visual stimuli contrast, that the retinotopy of classical attentional control areas in the parietal and frontal cortices was mainly driven by attention, and that the retinotopy of lateral temporal regions was driven by both. In a recent study, Bressler et al. (2013) measured the effects of endogenous visual spatial attention (i.e., attention directed voluntarily by the participant) on the amplitude of retinotopic responses in the occipital and parietal cortices. Participants were asked to direct their attention toward a target of different eccentricities and to detect a target during retinotopic mapping. The authors showed that attending to the target in the visual field enhanced the amplitude of activations in corresponding retinotopic cortical locations for all the areas investigated, but that the modulation of retinotopic responses depended on target eccentricity. In occipital areas (V1, V2, V3, and hV4), directed attention elicited greater activation in cortical locations which corresponded to target eccentricities closer to the center than those which were farther out. Conversely, in parietal areas, directed attention elicited greater activation in target eccentricities which were farther away than in those which were closer. The authors suggest that endogenous attention potentially plays a role in processing the fine details of an object in central vision and in detecting relevant objects in the periphery during motor planning. Interestingly, Sasaki et al. (2001) provided direct evidence of retinotopic modulation of response resulting from global and local attentional demands in the occipital cortex. The authors used very large hierarchical arithmetic symbols (for example, a global “x” form composed of several local “+” elements). During “attention to global” periods, participants focused their attention on the global symbol (the “x”) involving their peripheral vision, and during “attention to local” periods, they were instructed to focus attention on local symbols (the “+”), involving foveal vision. Results showed that when attention was directed at local (as opposed to global) level, activation occurred in the visual areas in relation to the foveal representation. When attention was directed at global (as opposed to local) level, activation was consistent with peripheral cortical representation. Since it can be assumed that global processing is mediated by low-pass spatial analysis, and local processing is mediated by high-pass spatial analysis (Schulman et al., 1986; Badcock et al., 1990; Lamb and Yund, 1993), the retinotopic organization observed in global and local attentional processing may constitute an argument in favor of a retinotopic organization for the attentional selection of spatial frequencies.

On the whole, the neuroimaging studies mentioned previously either highlight retinotopic mapping of spatial frequency processing (Sasaki et al., 2001), or reveal hemispheric specialization for spatial frequency processing (Iidaka et al., 2004; Peyrin et al., 2004). A recent fMRI study showed that spatial frequency processing could be both retinotopically mapped and lateralized between the two hemispheres (Musel et al., 2013).

### RETINOTOPIC AND LATERALIZED PROCESSING OF SPATIAL FREQUENCIES DURING SCENE CATEGORIZATION

After demonstrating retinotopic organization of spatial frequency processing, Sasaki et al. (2001) concluded that neither global nor local processing was lateralized in the occipital cortex. However, the authors compared activation elicited by global and local conditions to one another (traditional method of fMRI data analysis), rather than activation between hemispheres (direct inter-hemispheric comparison method used in Peyrin et al., 2004). Musel et al. (2013) evaluated both the retinotopy and the functional lateralization of spatial frequency processing using a categorization task of scenes (indoors vs. outdoors) filtered in HSF and LSF. They used larger scene images (with a visual angle of 24° × 18°) than in Peyrin et al. (2004) in which the visual angle was 4° × 4°, thus covering the same breadth of visual field as Sasaki et al. (2001). Results provided firstly evidence of retinotopic processing of spatial frequencies. At group level, the comparison between the spatial frequency content revealed that LSF scene categorization (as opposed to HSF) elicited activation in the anterior half of the calcarine fissures linked to the peripheral visual field, whereas HSF scene categorization (as opposed to LSF) elicited activation in the posterior part of the occipital lobes which are linked to the fovea, according to the retinotopic property of visual areas (**Figure 4B**). The retinotopic organization of spatial frequencies was also assessed at individual level by projecting LSF and HSF related activations onto retinotopic maps established for a number of participants. Functional activations projected onto individual retinotopic maps revealed that LSF processing is mapped in the anterior part of V1, whereas HSF processing is mapped in the posterior and ventral part of V2, V3, and V4. Furthermore, at the group level, the direct inter-hemispheric comparisons performed on the same fMRI data revealed a right-sided occipito-temporal predominance for LSF scene categorization and a left-sided temporal cortex predominance for HSF scene categorization, according to the hemispheric specialization theories. By using suitable method of fMRI analysis on the same data, as well as visual stimuli filtered in spatial frequencies covering a large part of the visual field, Musel et al. (2013) demonstrated for the first time retinotopic and lateralized spatial frequency processing in the human occipito-temporal cortex. It should be noted that hemispheric asymmetries were also highlighted within retinotopically defined parietal and frontal cortices during spatial working memory tasks (Sheremata et al., 2010; Szczepanski et al., 2010; Szczepanski and Kastner, 2013).

However, results from certain neurophysiological, computational, and behavioral studies indicate that the totality of spatial frequency information is not immediately conveyed through the brain, but that analysis follows a predominantly coarse-to-fine processing sequence. If LSF extraction and analysis occurs first, followed by that of HSF, why should there be any hemispheric lateralization for the processing of LSF or HSF? Identification of the neural basis of the coarse-to-fine analysis in scene perception is the first step toward responding to this question.

## COARSE-TO-FINE PROCESSING DURING SCENE PERCEPTION

### PSYCHOPHYSICAL ARGUMENTS OF COARSE-TO-FINE PROCESSING

Data from the functional neuroanatomy of magnocellular and parvocellular visual pathways indicate that the totality of visual information is not conveyed immediately, but that LSF reach the visual cortex before HSF (Van Essen and Deyoe, 1995; Bullier, 2001), although some controversies still remain (Merigan and Maunsell, 1993; Kaplan, 2004). A temporal precedence of LSF processing over HSF has been observed in psychophysical studies using sinusoidal gratings (Breitmeyer, 1975; Ginsburg, 1986; Hughes et al., 1996). Studies manipulating spatial frequency content of faces and scenes have provided further evidence of a coarse-to-fine processing sequence (Schyns and Oliva, 1994, 1997, 1999; Parker et al., 1996; Oliva and Schyns, 1997; Musel et al., 2012). Schyns and Oliva (1994) used hybrid images made of two superimposed scenes belonging to different categories and containing different spatial frequency bands (e.g., a city scene in LSF superimposed on a highway scene in HSF). When presentation time of hybrids was very short (30 ms), categorization of the hybrid was dominated by LSF information. However, categorization was dominated by HSF information for longer presentation times (150 ms). This suggests that LSF take precedence over HSF during scene perception. Furthermore, when the authors displayed two successive hybrids depicting simultaneously a coarse-to-fine sequence for a given scene (a LSF city in the first hybrid follows by a HSF city in the second hybrid) and a fine-to-coarse sequence for another scene (a HSF highway in the first hybrid follows by a LSF highway in the second hybrid), scene categorization was more frequently based on the coarse-to-fine than the fine-to-coarse sequence.

Although LSF information may be perceptually available before HSF, it is important to note that it does not necessarily follow that it is always used first to support visual recognition in all tasks. In Schyns and Oliva (1994), scene categorization in hybrid sequences was in fact based on a fine-to-coarse rather than a coarse-to-fine sequence in a substantial proportion of sequences (29%). Despite the apparent predominance of coarse-to-fine processing, certain flexibility in the processing sequence of spatial scale information has emerged, and it has also been seen to be sensitive to the demands of the task or the visual characteristics available in the stimuli (Parker et al., 1996; Schyns and Oliva, 1997, 1999; Morrison and Schyns, 2001; Mermillod et al., 2005; Ozgen et al., 2005, 2006; Rotshtein et al., 2010; Awasthi et al., 2013). A study by Schyns and Oliva (1999) showed that it was possible to constrain the spatial frequency band preferentially processed in hybrids by imposing a sensitization phase which implicitly “primes” visual processing in favor of a particular scale (coarse or fine). When participants were initially exposed to LSF information, subsequent categorization of hybrid images was preferentially performed following LSF cues, whereas it was biased toward HSF information after priming by HSF. The use of hybrid faces allowed Schyns and Oliva (1999) to show preferential recourse to HSF information to determine whether a face was expressive or not, and preferential recourse to LSF information to determine the nature of the emotion (e.g., happy, angry). It is therefore possible that the demands of a visual task determine which scale must be processed in hybrids (even using very short presentation). Overall, these studies suggest that all spatial frequencies are available at the beginning of categorization, and that their selection may depend on interactions between the perceptual information available and the demands of a given visual task.

Importantly, results from Schyns and Oliva (1994) studies suggest that coarse-to-fine processing constitutes a predominant and default strategy that seems advantageous for scene recognition (in the absence of task demands which constrain the use of a particular spatial frequency band). A recent study also evidenced a coarse-to-fine preference in the very early stages of development, in 7- to 8-months-old infants (Otsuka et al., 2014). Furthermore, a considerable number of recent studies have provided behavioral evidence of anLSF-based processing during rapid scene recognition (Kihara and Takeda, 2010; De Cesarei and Loftus, 2011; Musel et al., 2012; Mu and Li, 2013) and object categorization (Loftus and Harley, 2004). Using dynamic scenes composed of six filtered images of the same scene, from LSF to HSF or from HSF to LSF, allowing to experimentally mimic a coarse-to-fine or a reverse fine-to-coarse sequence, Musel et al. (2012) showed that coarse-to-fine sequences were categorized more rapidly than fine-to-coarse sequences in young adults. This provided new arguments in favor of a predominantly coarse-to-fine categorization of natural scenes, and a new experimental tool which imposes a coarse-to-fine processing and allows investigations of the neural substrates of coarse-to-fine processing.

### NEURAL BASIS OF COARSE-TO-FINE ANALYSIS

We do not as yet know exactly how and where in the brain LSF and HSF information is differentially analyzed and eventually merged during visual processing. Traditional models generally maintain that incoming visual cues are combined at successive stages along the cortical hierarchy (Biederman, 1995; Riesenhuber and Poggio, 1999), and suggest that LSF and HSF converge only in higher-level visual areas of the inferior temporal cortex (such as the fusiform or parahippocampal cortex; Bar et al., 2006). However, drawing on evidence obtained from neurophysiological recordings in nonhuman primates (Hupe et al., 2001), Bullier (2001) postulated that a rapid LSF analysis takes place predominantly in the dorsal cortical stream. Information is then sent-back through feedback signals into low-level areas (e.g., the primary visual cortex, V1), where it influences subsequent slower HSF analysis and guides subsequent processing through the ventral cortical stream. The occipital cortex might therefore serve as an “active blackboard” integrating computations made by higher-order cortical areas.

Bar et al. (2006) later investigated the neural correlates and time course of spatial frequency processing during object recognition in a combined fMRI and MEG study. They found evidence that stimuli containing LSF information elicited rapid activation in the orbitofrontal cortex, 50 ms before the involvement of recognition-related areas in the temporal cortex (fusiform gyrus). Activation of the orbitofrontal cortex was not observed with stimuli containing only HSF information. These authors suggested that the orbitofrontal cortex – mediated by LSF information – acts as the trigger of top-down facilitation during object recognition. Using dynamic causal modeling to investigate the interaction between the orbitofrontal cortex and the fusiform gyrus during the perception of LSF and HSF objects, Kveraga et al. (2007) showed reciprocal connections between these two cortical structures, with LSF modulating feedback connections from the orbitofrontal cortex to the fusiform gyrus. LSF may therefore reach the orbitofrontal cortex rapidly, in order to trigger plausible interpretations of any given visual input. The result of these computations would then be projected, via feedback connections, to the fusiform gyrus, and would guide subsequent analysis of HSF information. It is worth noting that in a recent study, Patai et al. (2013) presented LSF or HSF scenes as memory-cues (i.e., contextual information) and then asked participants to detect a target (e.g., an object) in the non-filtered version of the cued scene. These authors evidenced that LSF and HSF memory-cues were equally effective as triggers of contextual memory information, and facilitated target detection. This challenges Bar’s proposal of LSF-based facilitation in object recognition. However, their target detection task may have involved fine-grained perception, thus favoring HSF processing.

However, to date, the neural architecture and temporal dynamics of such top-down mechanisms have never been systematically investigated via direct testing of the preferential coarse-to-fine processing sequence during visual scene perception in humans. Peyrin et al. (2010) combined fMRI and ERPs on the same participants to identify the neural substrates underlying the coarse-to-fine processing sequence. To constrain the order of spatial frequency processing, the authors displayed sequences of two spatial frequency-filtered scenes in rapid succession, with either a coarse-to-fine sequence (LSF scene followed by a HSF scene), or a fine-to-coarse sequence (HSF scene followed by an LSF scene). Participants’ task was to decide whether the two scenes belonged to a same category (city, beach, or indoor). FMRI examination revealed selective increased activation in early stage occipital areas, and in frontal and tempo-parietal areas for coarse-to-fine sequences (compared to fine-to-coarse sequences). ERP topography and source analyses revealed a similar cortical network, but could additionally determine the time-course of activation in these areas. Frontal and temporo-parietal areas responded more to LSF scenes when these were presented first, whereas the occipital areas responded more to HSF scenes when these were presented after LSF scenes. More specifically, results demonstrated that low-pass signals (conveyed by fast magnocellular pathways) could rapidly activate high-order areas, providing semantic information (via the left prefrontal cortex and temporal areas) and spatial information (via the frontal eye fields), as well as attentional controls (via the temporo-parietal junction), all of which may promote the ongoing categorization and perceptual organization of the scene. This low-pass or coarse analysis is perhaps refined by further processing of high-pass signals (conveyed more slowly by the parvocellular pathways). To enable this, feedback from the low-pass analysis, which take place in frontal and temporo-parietal areas, might be sent back into lower level visual areas, such as the primary visual cortex, and would then guide the high-pass analysis and assist in the selection of the relevant finer details necessary for the recognition and categorization of scenes. These results are consistent with the LSF-based top-down facilitation of recognition, as proposed by Bar et al. (2006; see also Bar, 2003) in the context of object recognition, with the exception of the cortical site for feedback projections (occipital cortex in Peyrin et al., 2010; fusiform gyrus in Bar et al., 2006).

The influential models of visual perception assume a predominantly coarse-to-fine sequence of spatial frequency processing in the whole brain, based on the functional properties of the visual pathways. However, as mentioned previously, many studies have also shown that it is possible that the two hemispheres of the human brain may complement one another in the processing of LSF and HSF. The critical issue here is how to reconcile hemispheric specialization of spatial frequency processing with coarse-to-fine analysis of scenes.

### CEREBRAL ASYMMETRIES FOR COARSE-TO-FINE PROCESSING

The hemispheric specialization observed for spatial frequency processing raise the crucial question of the legitimacy of suggesting that coarse-to-fine sequencing is applied throughout brain. Peyrin et al. (2005) conducted an fMRI experiment in order to investigate whether coarse-to-fine processing predominates in only one hemisphere. They displayed sequences of two spatial frequency-filtered scenes in rapid succession, with either a coarse-to-fine sequence (LSF scene followed by HSF scene), or a fine-to-coarse sequence (HSF scene followed by LSF scene). Participants’ task was to decide whether the two scenes belonged to a same category (city, beach, or indoor). Cerebral asymmetries were identified using inter-hemispheric method of comparison (i.e., contrast between “unflipped” and “left-right flipped” functional images for each sequence). Results showed greater activation in the right than the left occipito-temporal cortex for the coarse-to-fine sequence, and greater activation in the left than the right occipito-temporal cortex for the fine-to-coarse sequence. These fMRI results suggest that the initial spatial frequency-band appearing in the sequence could determine which of the two hemispheres is preferentially involved in the sequential processing of spatial frequencies. According to input sequences or task demands, the right occipital cortex would give priority to LSF analysis for a coarse-to-fine processing and the left occipital cortex would give priority to HSF analysis for a fine-to-coarse analysis.

As far as the higher-level stages of visual scene processing are concerned, several studies have highlighted the sensitivity of scene-selective areas to low-level features, such as spatial frequencies and amplitude spectrum properties, in scenes (Andrews et al., 2010; Rajimehr et al., 2011; Zeidman et al., 2012). However, we still lack evidence of coarse-to-fine processing within the scene-selective cortical regions.

## SPATIAL FREQUENCY PROCESSING WITHIN SCENE-SELECTIVE AREAS

There is considerable evidence suggesting that the occipito-temporal cortex contains a mosaic of different areas that respond selectively to different category of stimuli (Haxby et al., 2001; Lerner et al., 2001; Spiridon and Kanwisher, 2002). More specifically, three regions were evidenced as scene-selective regions: the PPA, the RSC, and the OPA. These regions are known to be involved in high-level functions such as navigation (Epstein et al., 2007; Vass and Epstein, 2013), spatial layout processing and scene recognition (Epstein and Kanwisher, 1998; Epstein et al., 1999, 2003; Epstein, 2005, 2008; Epstein and Higgins, 2007; Epstein and Ward, 2010; Dilks et al., 2013), and contextual associations (Bar and Aminoff, 2003; Bar, 2004; Aminoff et al., 2007; Bar et al., 2008a,b). However, only a few studies investigated whether these regions are sensitive to scenes low-level properties such as spatial frequencies. For example, Peyrin et al. (2004) showed that the parahipopcampal gyrus was more strongly activated by LSF than HSF scenes. Conversely, Rajimehr et al. (2011) observed that in human and macaques, the PPA responded more strongly to HSF than LSF stimuli. This was also the main findings of Zeidman et al. (2012). In their study, they depicted three-dimensional spaces by positioning small dots following an exponential distribution and filtered them in either LSF or HSF. They showed stronger activation of the PPA when participants had to detect de disappearance of a small proportion of dots in HSF than LSF spaces. It should be noted that these studies differed in many methodological aspects such as the task demands or the duration of stimuli, that may have influenced spatial frequency selectivity within the PPA. However, whether coarse-to-fine processing of scenes occurs within scene-selective regions is still unclear.

Coarse-to-fine processing of faces in high level visual cortex was the central focus of a recent study by Goffaux et al. (2011) who showed an intriguing effect of spatial frequencies in a face-selective region, the fusiform face area (FFA; Kanwisher et al., 1997). By manipulating duration of exposure and the spatial frequency content of faces, these authors observed higher levels of FFA response to LSF when duration of exposure to faces was short, and higher levels of response to HSF for longer exposure durations. These results suggest that coarse-to-fine processing is the predominant strategy in the most prominent regions of the ventral visual stream (inferotemporal cortex). In an evoked potential study, Schettino et al. (2011) used sequences of filtered scenes (with blank screens occurring between scenes) in order to investigate the neural correlates of the accumulation of visual information during object recognition and the time course of these correlates. For this purpose, the authors used sequences in which the first scene was always in LSF and the scene was gradually revealed in six successive images by progressively adding HSF information. The authors observed that activation in the parahippocampal cortex decreases when the spatial frequency content of scenes increases, suggesting that this region is sensitive to the primary processing of LSF information, even if this study did not investigate explicit coarse-to-fine processing of scenes.

**FIGURE 5:**
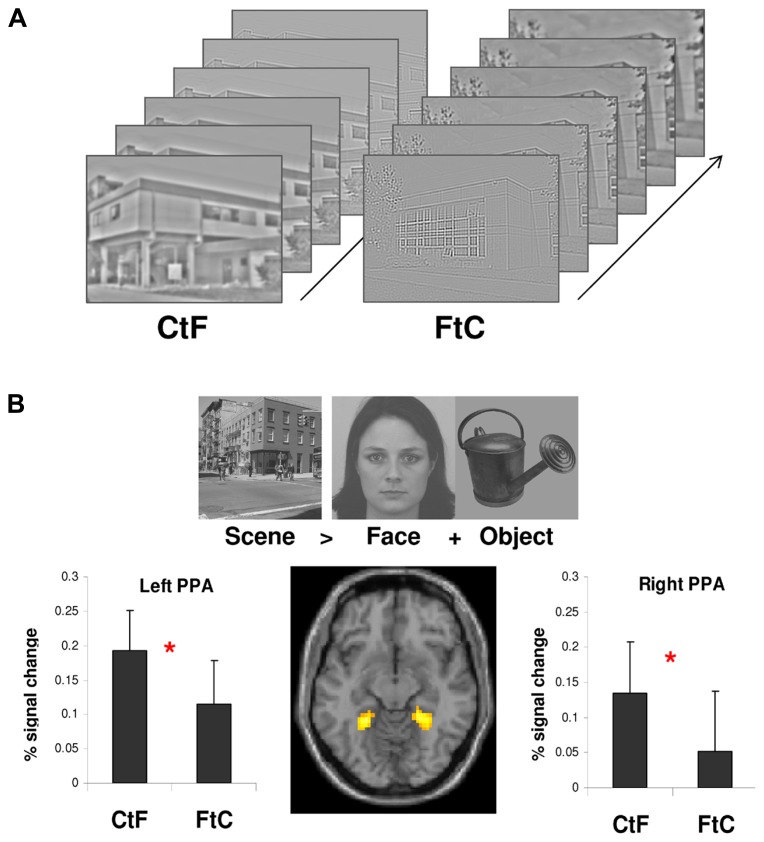
**(A)** Six spatial frequency filtered images of scenes that depict a coarse-to-fine (CtF) and fine-to-coarse (FtC) sequences. **(B)** Parahippocampal place area (PPA) localized by contrasting the activation induced by the perception of scenes to those induced by the perception of faces and objects. Signal changes relative to the global mean intensity of signal were then extracted from the PPAs for each sequence (CtF and FtC). PPA showed stronger activation during the CtF than FtC categorization of sequences. Error bars indicate 95% Confidence Intervals. * Indicate significant differences. Figure adapted from Musel et al. (2014).

A recent fMRI study (Musel et al., 2014) tested whether such processing occurs in three scene-selective cortical regions: the PPA, the RSC, and the OPA. We measured activation in these scene-preferring regions during the categorization of dynamic scene stimuli (Musel et al., 2012). Dynamic scenes were composed of six filtered images of the same scene, from LSF to HSF or from HSF to LSF, allowing us to mimic either a coarse-to-fine or a fine-to-coarse sequence. We first identified scene-selective regions using a localizer adapted from previous studies (Epstein and Kanwisher, 1998; Epstein et al., 2003; Bar et al., 2008b; Walther et al., 2009) in which participants viewed gray-scale photographs of scenes, faces and common objects. The contrast between scenes and other categories was intended to enable localization of the regions involved in the perception of scenes. Once localized, we compared activation elicited by coarse-to-fine and fine-to-coarse dynamic scenes within the areas defined as the PPA, RSC, and OPA. Results showed greater activation of only the PPA for coarse-to-fine compared to fine-to-coarse sequences (**Figure 5**). Equivalent activations were observed for both types of sequence in the RSC and OPA. This study therefore suggests that coarse-to-fine sequence processing constitutes the predominant strategy for scene categorization in the PPA. It should be noted that evidence of spatial frequency sensitivity within other scene-selective areas, such as the RSC and the OPA, is still lacking.

## CONCLUSION

The present review aimed to identify cerebral regions differentially involved in low and high spatial frequency processing and to clarify their attributes during scene perception. Several neuroimaging studies suggest that spatial frequency processing could be retinotopically mapped and lateralized in both hemispheres. Right occipital areas are more activated than the left ones during the processing of LSF scenes, while left occipital areas are more activated than the right ones during the processing of HSF scenes. Concomitantly, the processing of HSF scenes (as opposed to LSF) activates the foveal representation in retinotopic areas of the occipital cortex, and LSF scenes (as opposed to HSF) activate more peripheral representations in retinotopic areas.

The present review also studied the neural bases of coarse-to-fine analysis as a default and predominant processing strategy. According to influential models (Bullier, 2001; Bar, 2003; Bar et al., 2006; Kveraga et al., 2007; Peyrin et al., 2010), LSF information may reach high-order areas rapidly, enabling coarse initial parsing of the visual scene, which can then be sent back through feedback connections into lower level visual areas to guide a finer analysis based on HSF. Studies also indicate that in scene perception, coarse-to-fine processing seems to be preferentially performed in the right hemisphere, from the occipital to the inferior temporal cortex. Overall, results from neuroimaging studies are consistent with the idea that explicit vision advances in a reverse hierarchical direction, as hypothesized by Hochstein and Ahissar (2002) and Ahissar and Hochstein (2004; see The Reverse Hierarchy Theory). According to this theory, rapid visual perception is not purely feedforward, it is also strongly mediated by top-down influences by high-level areas on lower-level areas. Finally, the present review addressed spatial frequency processing within scene-selective cortical areas. We reported results demonstrated that the coarse-to-fine strategy is a plausible modus operandi in the PPA.

**FIGURE 6:**
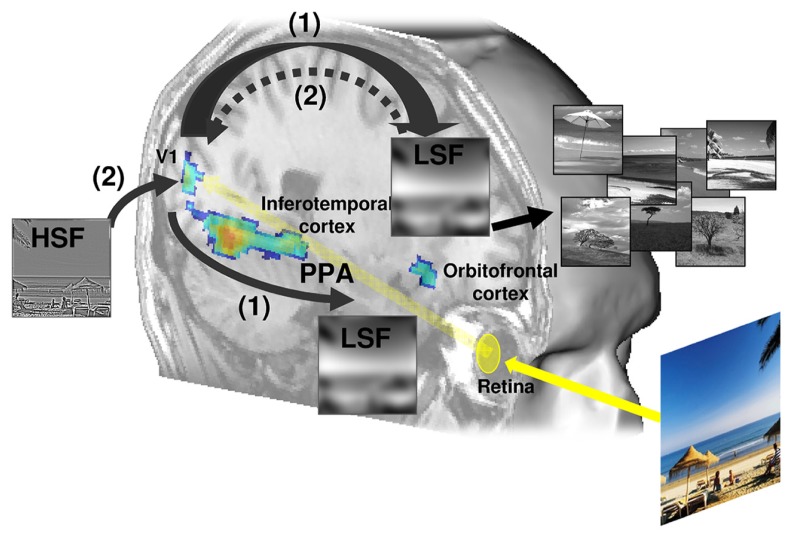
**A schematic illustration of the proposed coarse-to-fine cortical model.** (1) Los spatial frequency (LSF) information reaches high-order areas of the dorsal visual stream rapidly, enabling coarse initial parsing of the visual scene (providing the spatial organization of the scene through the frontal eye fields and possible interpretations of the category of the scene through the orbitofrontal cortex), prior to its complete propagation along the ventral visual stream (inferotemporal cortex) that ultimately mediates the scene recognition. (2) This initial low-pass analysis might be then “retro-injected” through feedback into lower level areas (including the primary visual cortex, V1) to guide a slower analysis of high spatial frequency (HSF) information through the ventral visual stream and select the relevant finer details necessary for the recognition and identification. In this model, the coarse-to-fine analysis is preferentially performed in the right ventral visual stream, from the occipital to the inferior temporal cortex and the parahippocampal place area (PPA).

Overall, these results obviously raised the question of the connectivity between the PPA and the cortical network specifically involved in coarse-to-fine processing. Baldassano et al. (2013) recently demonstrated that the PPA exhibits a gradient in connectivity with other scene-specific regions along the anterior-posterior axis in a way that suggests that the posterior part of the PPA is more closely connected to occipital areas and therefore contributes more to the processing of low level visual features (possibly to spatial frequencies and spatial envelope properties) while the anterior part of the PPA is more closely connected to the RSC and therefore contributes to the construction of a global scene representation. In Musel et al. (2014), the contrast between coarse-to-fine and fine-to-coarse processing revealed significant activation within the orbitofrontal cortex and the primary visual cortex (**Figure 6**). These two regions might play a predominant role during the coarse-to-fine categorization of scenes. Involvement of the orbitofrontal cortex was previously evidenced in rapid LSF-based categorical inferences (Bar et al., 2006; Peyrin et al., 2010) and the primary visual cortex was evidenced to be one of the cortical sites in which the first LSF computation could be “retro-injected” to guide the subsequent finer analysis of HSF (Bullier, 2001; Peyrin et al., 2010). In a proactive brain model, Bar (2007) attempts to clarify the functional role of the parahippocampal cortex (including the PPA) in object recognition. According to this model, LSF information in an object is projected from early stage visual areas to the orbitofrontal cortex. Based on the global appearance of the object, this region then triggers activation of the most probable object identities. Parallel projection of LSF information to the parahippocampal cortex and the PPA also occurs to extract the context in which this object appears and activates its contextual associations. The intersection of possible object identities (from the orbitofrontal cortex) and the objects that typically appear in such contexts (from the parahippocampal cortex) provides fast and coarse recognition of the current view of the object. This assumption is supported by studies on the macaque brain which indicate that the orbitofrontal cortex has strong and reciprocal links with the temporal cortex, notably medial regions including parahippocampal areas (Cavada et al., 2000). In humans, studies using diffusion tensor MRI have evidenced structural connectivity between the parahippocampal cortex and orbitofrontal areas (Powell et al., 2004). Bar et al. (2006) also demonstrated strong synchrony between the orbitofrontal cortex and the temporal cortex during the recognition of LSF-filtered objects, suggesting important functional interactions between these regions. Unfortunately, to our knowledge, the functional connectivity or direct influence between the orbitofrontal cortex and PPA has not been demonstrated yet.

To conclude, the results reported in the present review provide critical support for influential models of visual perception mainly based on a spatial frequency analysis which follows a coarse-to-fine strategy (Schyns and Oliva, 1994; Bar, 2003; Hegde, 2008; Peyrin et al., 2010).

## Conflict of Interest Statement

The authors declare that the research was conducted in the absence of any commercial or financial relationships that could be construed as a potential conflict of interest.
